# Supplementary material for: Imaging the real space structure of the spin fluctuations in an iron-based superconductor
Source: Nat Commun. 2017 Jun 29;8:15996. doi: 10.1038/ncomms15996 (PMC5493767; doi:10.1038/ncomms15996)
Supplement: Supplementary Information [file ncomms15996-s1.pdf]

File name: Supplementary Information
Description: Supplementary Notes, Supplementary Figures and Supplementary References

File name: Peer Review File
Description:

Supplementary Note 1. CALCULATION OF THE SPIN SUSCEPTIBILITY AND ITS REAL SPACE STRUCTURE

We calculate the momentum-space structure of the spin susceptibility using a five band tight-binding model for LiFeAs[1,2] which is given by the Hamiltonian

$$H_{\mathrm{TB}} = \sum_{\mathbf{R},\mathbf{R}'\sigma} t^{\mu\nu}_{\mathbf{R},\mathbf{R}'} c^{\dagger}_{\mathbf{R}\mu\sigma} c_{\mathbf{R}'\nu\sigma}, \tag{1}$$

where $c^{\dagger}_{\mathbf{R}\sigma}$ is the creation operator of an electron, and $t^{\mu\nu}_{\mathbf{R},\mathbf{R}'}$ are hopping elements between orbitals μ and ν on Fe sites labeled with $\mathbf{R}$ and $\mathbf{R}'$. The corresponding Bloch Hamiltonian in momentum space reads

$$\hat{H}(\mathbf{k})^{\mu\nu} = \sum_{\delta} e^{i\mathbf{k}\cdot\delta} t^{\mu\nu}_{\delta}, \tag{2}$$

where $\delta = \mathbf{R} - \mathbf{R}'$ is the real-space distance of the hopping $t^{\mu\nu}_{\mathbf{R},\mathbf{R}'}$. The Hamiltonian matrix $\hat{H}(\mathbf{k})^{\mu\nu}$ has the eigenstates $a^{\ell}_{\mu}(\mathbf{k})$ with eigenenergies $\epsilon_{\mu}(\mathbf{k})$ such that the orbital susceptibility tensor in the normal state can be calculated by

$$\chi^0_{\ell_1\ell_2\ell_3\ell_4}(q) = -\sum_{k,\mu\nu} M^{\mu\nu}_{\ell_1\ell_2\ell_3\ell_4}(\mathbf{k},\mathbf{q}) G^{\mu}(k+q) G^{\nu}(k), \tag{3}$$

where we have adopted the shorthand notation of momentum space and Matsubara frequencies as $k \equiv (\mathbf{k}, \omega_n)$, and defined the matrix

$$M^{\mu\nu}_{\ell_1\ell_2\ell_3\ell_4}(\mathbf{k},\mathbf{q}) = a^{\ell_4}_{\nu}(\mathbf{k}) a^{\ell_2,*}_{\nu}(\mathbf{k}) a^{\ell_1}_{\mu}(\mathbf{k}+\mathbf{q}) a^{\ell_3,*}_{\mu}(\mathbf{k}+\mathbf{q}), \tag{4}$$

and the normal state Green functions $G^{\mu}(k) = [i\omega_n - \epsilon_{\mu}(\mathbf{k})]^{-1}$. Taking into account the superconducting order parameter on the bands crossing the Fermi level, $\Delta_{\mu}(\mathbf{k})$ approximated away from the Fermi surface by multiplying with a Gaussian damping[3,4], we obtain the susceptibility in the superconducting state as

$$\chi^0_{\ell_1\ell_2\ell_3\ell_4}(q) = -\sum_{k,\mu\nu} M^{\mu\nu}_{\ell_1\ell_2\ell_3\ell_4}(\mathbf{k},\mathbf{q}) [G^{\mu}(k+q) G^{\nu}(k) + F^{\mu}(k+q) F^{\nu}(k)], \tag{5}$$

where the Green functions are now $G^{\mu}(k) = (i\omega_n + \epsilon_{\mu}(\mathbf{k}))[(i\omega_n)^2 - (\epsilon_{\mu}(\mathbf{k}) + \Delta_{\mu}(\mathbf{k}))^2]^{-1}$ and $F^{\mu}(k) = -\Delta_{\mu}(\mathbf{k})^*[(i\omega_n)^2 - (\epsilon_{\mu}(\mathbf{k}) + \Delta_{\mu}(\mathbf{k}))^2]^{-1}$. The Matsubara sums can be carried out analytically such that the remaining task is the numerical calculation of the momentum space integrals working on the real frequency axis with a finite broadening of $\eta = 1$meV.

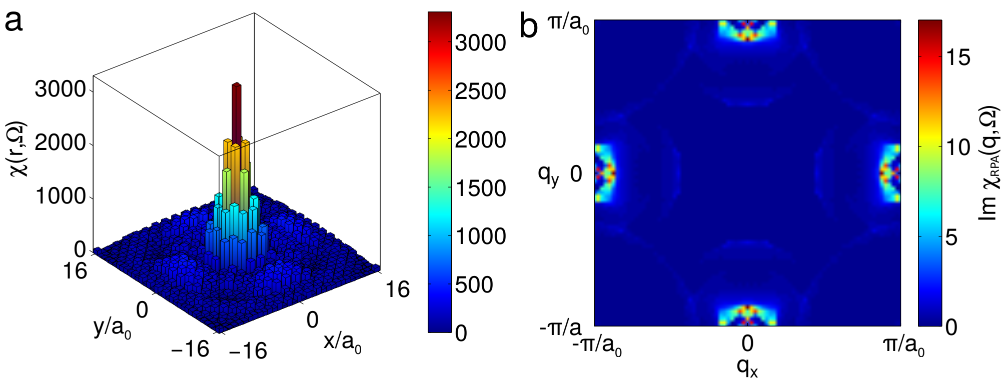

Supplementary Fig. 1: **Real space image of the spin fluctuations.** (**a**) Real space structure of the imaginary part of the spin susceptibility $\mathrm{Im}\chi(\mathbf{r},\Omega)$ at the energy of the spin-resonance mode with $\Omega \approx 2\Delta$, where Δ is the size of the superconducting gap and Ω the energy of the spin resonance. (**b**) Momentum space structure of the spin susceptibility $\mathrm{Im}\chi_{\mathrm{RPA}}(\mathbf{q},\Omega)$ at the same energy as in **a**.

Taking into account interactions from a Hubbard-Hund Hamiltonian with $U = 0.93\,\mathrm{eV}$ and $J = U/4$ via the random phase approximation (RPA), we obtain the susceptibility as

$$\chi_{\mathrm{RPA}}(\mathbf{q},\omega) = \sum_{\ell_1,\ell 2} \left\{ \chi^0(q)\left[1 - \bar{U}\chi^0(q)\right]^{-1} \right\}_{\ell_1\ell_1\ell_2\ell_2}, \tag{6}$$

where $\bar{U}$ is a matrix containing the bare interactions. For the superconducting state we use a sign changing $s_\pm$ gap structure with anisotropies as shown in Ref. 1 and the same gap structure where the sign change has been removed artificially as discussed in the same reference. The real space structure of the imaginary part of the spin susceptibility $\mathrm{Im}\chi(\mathbf{r},\omega)$ is then calculated via

$$\mathrm{Im}\chi(\mathbf{r},\omega) = \int_{\mathbf{q}} \mathrm{Im}\,\chi_{\mathrm{RPA}}(\mathbf{q},\omega)\exp(-i\mathbf{q}\cdot\mathbf{r})\mathrm{d}\mathbf{q}, \tag{7}$$

such that the local susceptibility $\mathrm{Im}\chi(\omega) = \mathrm{Im}\chi(0,\omega)$ by definition, and the real space susceptibility as discussed in the main text is translationally invariant, e.g. $\mathrm{Im}\chi(\mathbf{r},\mathbf{r}',\omega) = \mathrm{Im}\chi(\mathbf{r}-\mathbf{r}',\omega)$. In Supplementary Figure 1a the real-space spin susceptibility in the superconducting state ($s_\pm$) is presented, which shows significant contributions at $\mathbf{r} \neq 0$ and some longer range oscillations. The value at $x = 0$ and $y = 0$ is the value of the local susceptibility at the resonance energy as discussed and shown in the main text. For completeness, we

show in Supplementary Figure 1b, the momentum space structure of the spin susceptibility $\mathrm{Im}\,\chi_{\mathrm{RPA}}(\mathbf{q},\Omega)$ at the resonance energy $\Omega \approx 2\Delta$.

Supplementary Note 2. CALCULATION OF THE DIFFERENTIAL CONDUCTANCE

For the simulation of the tunneling spectra including inelastic contributions, we use the band structure and superconducting gap as calculated and presented earlier[1,2] which yields agreement to the density of states (within the energy range of the order parameter) as measured in STM. Next, the spectral density of the spin fluctuations in the superconducting state is calculated[3,4] which after integrating over the $\mathbf{q}$-space following Supplementary Eq. 7 (for $\mathbf{r} = 0$) yields the result as shown in Fig. 1(c) and the inset of Fig. 2(a). Spectra as shown in Fig. 2(d) are obtained following Refs. 5–7, by calculating the inelastic contribution to the tunneling current from

$$
\begin{aligned}
g_{\mathrm{inel}}(V) \;\propto\; & \int_{-\infty}^{\infty} \mathrm{Im}\chi(\omega)\rho(\epsilon)\,\mathrm{d}\epsilon\,\mathrm{d}\omega \\
& [f'(\epsilon+\omega+eV)(1+n_{\mathrm{B}}(\epsilon))(1-f(\epsilon)) && (8)\\
& +f'(\epsilon-\omega+eV)n_{\mathrm{B}}(\omega)(1-f(\epsilon)) && (9)\\
& +f(\epsilon)(1+n_{\mathrm{B}}(\omega))f'(\epsilon-\omega+eV) && (10)\\
& +f(\epsilon)n_{\mathrm{B}}(\omega)f'(\epsilon+\omega+eV)]\,, && (11)
\end{aligned}
$$

where $\mathrm{Im}\chi(\omega)$ is the imaginary part of the spin susceptibility, $\rho(\omega)$ the density of states of the sample, $f(\omega)$ the Fermi function, $f'(\omega)$ the first derivative of the Fermi function and $n_{\mathrm{B}}(\omega)$ the Bose Einstein distribution. In the limit of $T \to 0$ and for the unoccupied states, this yields Eq. 1. The tunneling processes contributing to the inelastic current are shown in Supplementary Figure 2. To include the effect of a sharp impurity resonance (see Figure 2(e)), the density of states is modified by adding a Gaussian peak, which is very similar to the experimentally measured spectra, see Supplementary Figure 3(b)[1,8,9] and can also be obtained within a T-matrix calculation using a reasonably strong potential scatterer[1,10].

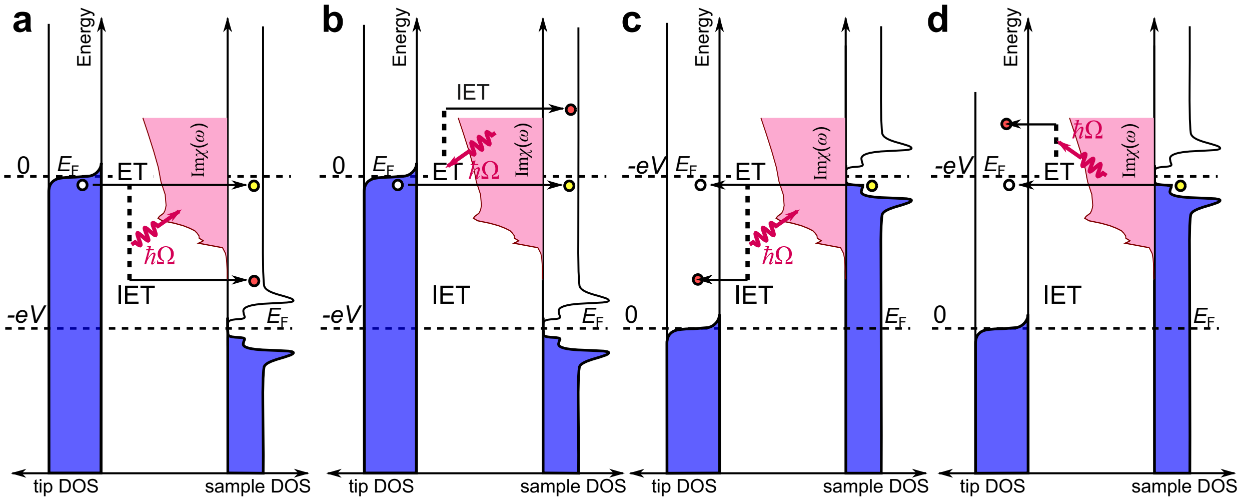

Supplementary Fig. 2: **Inelastic tunneling processes.** (**a**) Elastic (ET) and Inelastic (IET) tunneling processes for an electron tunneling from tip to sample. The inelastic process corresponds to supplementary equation 8 during which a spin excitation of energy Ω is emitted. The inelastic tunneling process effectively creates a replica feature of peaks in the density of states. (**b**) Process corresponding to Supplementary Equation 9, an excitation with energy Ω is absorbed during the tunneling process. (**c**, **d**) Schematics of the corresponding processes for an electron tunneling from sample to tip (Supplementary Equations 10, 11). Processes **b** and **d** are strongly suppressed at low temperatures, because $n_\mathrm{B}(\Omega)$ becomes zero for non-zero Ω.

Supplementary Note 3. DISTANCE DEPENDENCE OF REPLICA FEATURE

To isolate the features in the tunneling spectra due to defects, we consider difference spectra $\delta g_\mathrm{S}(r, V) = g_\mathrm{S}(r, V) - g_\mathrm{S}(r \to \infty, V)$ obtained through subtracting spectra obtained on the clean surface, $g_\mathrm{S}(r \to \infty, V)$, from all spectra $g_\mathrm{S}(r, V)$. As shown in Supplementary Fig. 4, in addition to the intensity decay over distance, the energy (bias voltage) of the maxima of the replica features also shifts with distance to the defects. However, the energies of the bound states barely depend on position. In this section, we introduce a simple model to show that the energy shift can largely be accounted for by the LDOS variation as a function of distance from the defects, and is thus unlikely an intrinsic feature of the spin excitations. For this model, we separate the influence of the replica feature, which is assumed to occur at a fixed energy, from the varying linear background of the tunneling

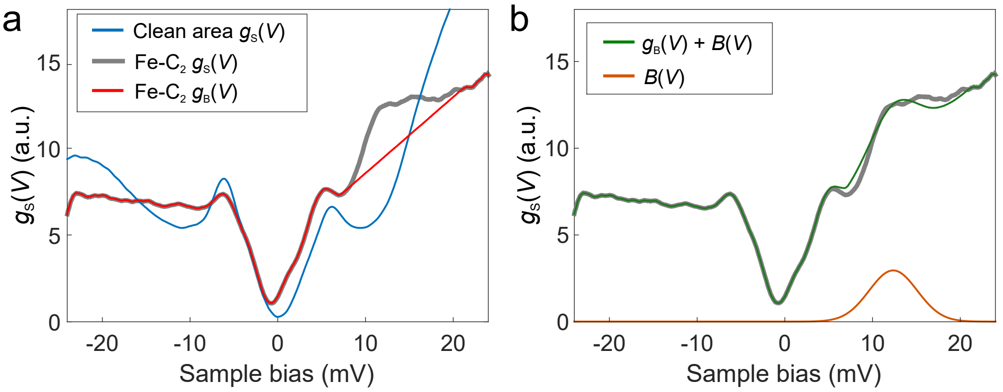

Supplementary Fig. 3: **Modeling of the replica feature.** (**a**) Differential conductance $g_S(V) = dI_t/dV_s$ on a clean area (blue) and a Fe-C_2 defect (gray) measured at $T = 4.2$ K. The red spectrum is the synthetic background spectrum $g_B(r = 0, V)$ on an Fe-C_2 defect (with the replica feature removed). (**b**) The Gaussian bump, $B(V)$ (orange) is used to represent the replica feature within the dip-hump energy range. The green spectrum $g_S^{\rm Sim}(r = 0, V)$ is a sum of the background Fe-C_2 spectrum in **a** and the Gaussian bump, reproducing the experimental spectrum (gray).

spectra. As shown in Supplementary Figure 3a, the replica feature resides in a bias range where the STS on both a clean area (blue) and a Fe-C_2 defect (gray) vary dramatically. In particular, the slope at this bias range becomes steeper with increasing distance from the Fe-C_2 defect. From Fig. 3a, we see that, in the normal state, the tunneling spectrum on the Fe-C_2 defect is featureless within the dip-hump bias voltage range. Thus, for simplicity, we define a background spectrum $g_B(r = 0, V)$ on the defect (here Fe-C_2) where the spectrum within the dip-hump energy range is assumed to be linear, (red spectrum in Supplementary Figure 3a), effectively removing the replica feature. A Gaussian bump, $B(V)$, is used to represent the replica feature. The total spectrum at the defect is then $g_S^{\rm Sim}(r = 0, V) = g_B(r = 0, V) + B(V)$. As shown in Supplementary Figure 3b, these assumptions reproduce the spectrum on the Fe-C_2 defect well.

To account for the distance dependence, we use the fits to the experimentally observed distance dependence of the replica feature, $h(r)$, and of the bound state, $d(r)$, shown in Fig. 5c. The background STS $g_B(r, V)$ is a weighted combination of the STS on the defect, $g_B(r = 0, V)$, and on the clean surface, $g_B(r \to \infty, V) = g_S(r \to \infty, V)$, the weight given by the experimentally observed spatial dependence of the bound state feature, $d(r)$. Therefore,

the background STS $g_{\rm B}(r,V)$ is calculated from

$$g_{\rm B}(r,V) = d(r)g_{\rm B}(r=0,V) + (1-d(r))g_{\rm S}(r\to\infty,V). \tag{12}$$

The tunneling spectrum at distance r is then given by

$$g_{\rm S}^{\rm Sim}(r,V) = g_{\rm B}(r,V) + h(r)B(V), \tag{13}$$

where the term $h(r)B(V)$ represents the distance dependence of the replica feature. The simulated difference $\delta g_{\rm S}^{\rm Sim}(r,V)$ is calculated by

$$\delta g_{\rm S}^{\rm Sim}(r,V) = g_{\rm S}^{\rm Sim}(r,V) - g_{\rm S}(r\to\infty,V). \tag{14}$$

Supplementary Figure 4 shows the comparison between the experimental $\delta g_{\rm S}(r,V)$ (panel a) and simulated $\delta g_{\rm S}^{\rm Sim}(r,V)$ (panel b) spectra. The simulation, which takes the slope of the background density of states into account, is in agreement with the experimental observation. Supplementary Figure 4c shows the bias voltage dependence of the maxima in $\delta g_{\rm S}(r,V)$ and $\delta g_{\rm S}^{\rm Sim}(r,V)$ on the distance away from the defect center. Near the defect center, because the defect is not point-like, the fits of exponential and $1/r$ decay do not work (compare Fig. 5). Our simulation starts from 0.5 nm away from the defect center, where the maximum of the feature intensity is. With this simple model, our simulation captures the major, if not all, contribution of the energy shift of the replica feature in the $\delta g_{\rm S}(r,V)$ spectra.

Supplementary Note 4. COMPARISON OF INELASTIC FEATURES FOR s_{++} AND $s_{\pm}$ ORDER PARAMETERS

In Supplementary Figure 5, we compare the simulated tunneling spectra (ET + IET) for different order parameters as well as with the experimental data. For the calculation with s_{++} and $s_{\pm}$ order parameters, the same modulus of the gap function is assumed. The gap function reproduces the experimentally observed double-gap structure with a smaller gap $\Delta_{\rm small}$ and a larger gap Δ. The spin susceptibility with only $s_{\pm}$ order parameter shows a resonance peak, which gives raise to the dip-hump structure in the simulated spectrum (blue in Supplementary Figure 5b). In the experimental data (Supplementary Figure 5c), above the superconducting gap, a dip at $\sim 2\Delta$ followed by a *kink* at $\sim 3\Delta$ appears in the superconducting spectrum, a feature only reproduced in the calculated spectrum for

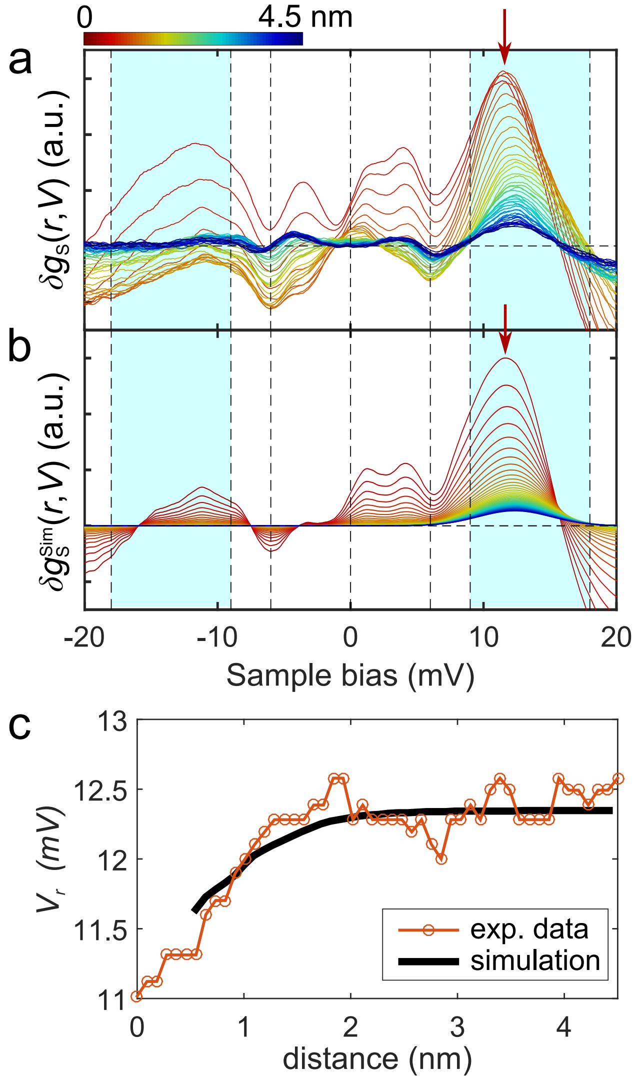

Supplementary Fig. 4: **Distance dependence of the energy of the replica feature.** (**a**) Experimental $\delta g_{\mathrm{S}}(r, V)$ as shown in Fig. 4a. (**b**) Simulated $\delta g_{\mathrm{S}}^{\mathrm{Sim}}(r, V)$ as obtained from the model (Supplementary Equation 14). The replica feature is indicated by the red arrows. **c**. Comparison of the bias voltage dependence of the maximum of the replica feature obtained experimentally with the one extracted from the model.

the $s_{\pm}$ order parameter. This *kink* feature locates at the energy of $\Delta + \Omega$, where Δ is the superconducting gap and Ω is the spin resonance energy.

In Supplementary Figure 6, the difference caused by superconducting order parameters is further examined in the normalized $g_{\mathrm{S}}(V)/g_{\mathrm{N}}(V)$ spectra. A dip-hump feature due to the resonance in the spin susceptibility is clearly found in the spectrum for the $s_{\pm}$ order

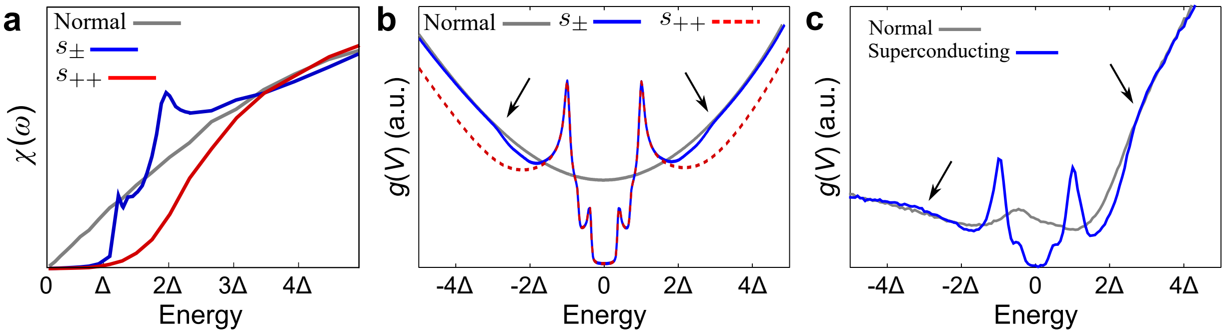

Supplementary Fig. 5: **Comparison of calculated tunneling spectra (ET + IET) with experimental data.** (**a**) The calculated the spin susceptibility in the normal state (gray), and the superconducting state with $s_\pm$ (blue) and s_{++} (red) order parameters. (**b**) The calculated spectra with the IET contribution. (**c**) Experimental spectra of the normal (gray) and superconducting (blue) states on a clean surface. Black arrows indicate the peculiar *kinks* in the calculated spectrum with the $s_\pm$ order parameter and experimental data.

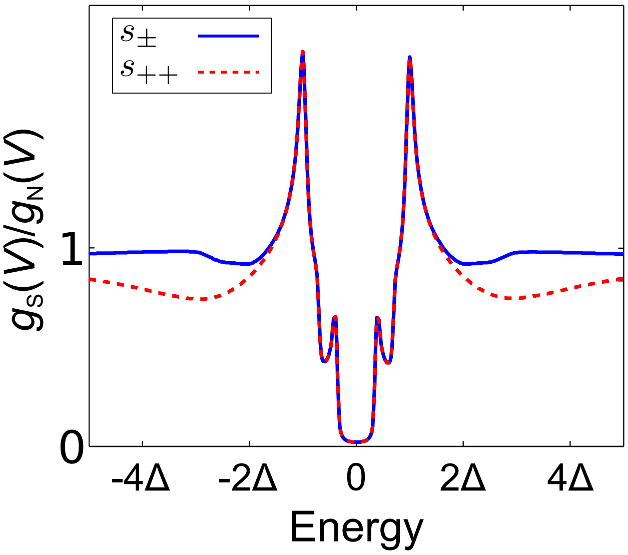

Supplementary Fig. 6: **Comparison of spectra for $s_\pm$ and s_{++} order parameters.** Calculated $g_S(V)/g_N(V)$ spectra for $s_\pm$ (blue) and s_{++} (red) order parameters.

parameter within $E \leq 3\Delta$, whereas only a featureless dip is seen in the spectrum for the s_{++} order parameter.

Supplementary References

1. Chi, S. *et al.* Impact of Iron-site defects on Superconductivity in LiFeAs. *Phys. Rev. B* **94**, 134515 (2016).
2. Kreisel, A. *et al.* Towards a quantitative description of tunneling conductance of superconductors: Application to LiFeAs. *Phys. Rev. B* **94**, 224518 (2016).
3. Maier, T. A., Graser, S., Scalapino, D. J. & Hirschfeld, P. Neutron scattering resonance and the iron-pnictide superconducting gap. *Phys. Rev. B* **79**, 134520 (2009).
4. Kreisel, A., Mukherjee, S., Hirschfeld, P. J. & Andersen, B. M. Spin excitations in a model of FeSe with orbital ordering. *Phys. Rev. B* **92**, 224515 (2015).
5. Kirtley, J. R. & Scalapino, D. J. Inelastic-tunneling model for the linear conductance background in the high-T_c superconductors. *Phys. Rev. Lett.* **65**, 798 (1990).
6. Kirtley, J. R. Inelastic transport through normal-metal – superconductor interfaces. *Phys. Rev. B* **47**, 11379–11383 (1993).
7. Hlobil, P., Jandke, J., Wulfhekel, W. & Schmalian, J. Tracing the electronic pairing glue in unconventional superconductors via inelastic Scanning Tunneling Spectroscopy. *Phys. Rev. Lett.* **118**, 167001 (2017).
8. Grothe, S. *et al.* Bound states of defects in superconducting LiFeAs studied by scanning tunneling spectroscopy. *Phys. Rev. B* **86**, 174503 (2012).
9. Schlegel, R. *et al.* Defect states in LiFeAs as seen by low-temperature scanning tunneling microscopy and spectroscopy. *phys. status solidi (b)* **254**, 1600159 (2017).
10. Gastiasoro, M. N., Hirschfeld, P. J. & Andersen, B. M. Impurity states and cooperative magnetic order in Fe-based superconductors. *Phys. Rev. B* **88**, 220509 (2013).
